# Perirenal Adipose Tissue from Healthy Donor: Characteristics and Promise as Potential Therapeutic Cell Source

**DOI:** 10.3390/jcm10215024

**Published:** 2021-10-28

**Authors:** Eun Hye Lee, So Young Chun, Jun Nyung Lee, Jae-Wook Chung, Bo Hyun Yoon, Hyun Tae Kim, Tae Gyun Kwon, Yun-Sok Ha, Bum Soo Kim

**Affiliations:** 1 Joint Institute for Regenerative Medicine, Kyungpook National University, Daegu 41405, Korea; eun90hye@gmail.com (E.H.L.); bobo1904@naver.com (B.H.Y.); 2 Biomedical Research Institute, Kyungpook National University Hospital, Daegu 41940, Korea; soyachun99@naver.com; 3 Department of Urology, School of Medicine, Kyungpook National University, Daegu 41566, Korea; ljnlover@gmail.com (J.N.L.); jeus119@hanmail.net (J.-W.C.); urologistk@knu.ac.kr (H.T.K.); tgkwon@knu.ac.kr (T.G.K.)

**Keywords:** perirenal, adipose tissue, healthy donor, brown adipocyte, beige cell

## Abstract

Perirenal adipose tissue, one of the fat masses surrounding the kidneys, can be obtained from healthy donors during a kidney transplant. Perirenal adipose tissue has only ever been known as a connective tissue to protect the kidneys and renal blood vessels from external physical stimulation. Yet, recently, as adipose tissue has begun to be considered an endocrine organ, and perirenal adipose tissue is now regarded to have a direct effect on metabolic diseases. The characteristics of perirenal adipose tissue from a healthy donor are that: (1) There are a large number of brown adipose cells (70–80% of the total), (2) Most of the brown adipose cells are inactive in the resting cell cycle, (3) Activating factors are constant low-temperature exposure, hormones, metastasis factors, and environmental factors, (4) Anatomically, a large number of brown adipose cells are distributed close to the adrenal glands, (5) Beige cells, produced by converting white adipocytes to brown-like adipocytes, are highly active, (6) Activated cells secrete BATokines, and (7) Energy consumption efficiency is high. Despite these advantages, all of the perirenal adipose tissue from a healthy donor is incinerated as medical waste. With a view to its use, this review discusses the brown adipocytes and beige cells in perirenal adipose tissue from a healthy donor, and proposes opportunities for their clinical application.

## 1. Perirenal Adipose Tissue

**Figure 1:**
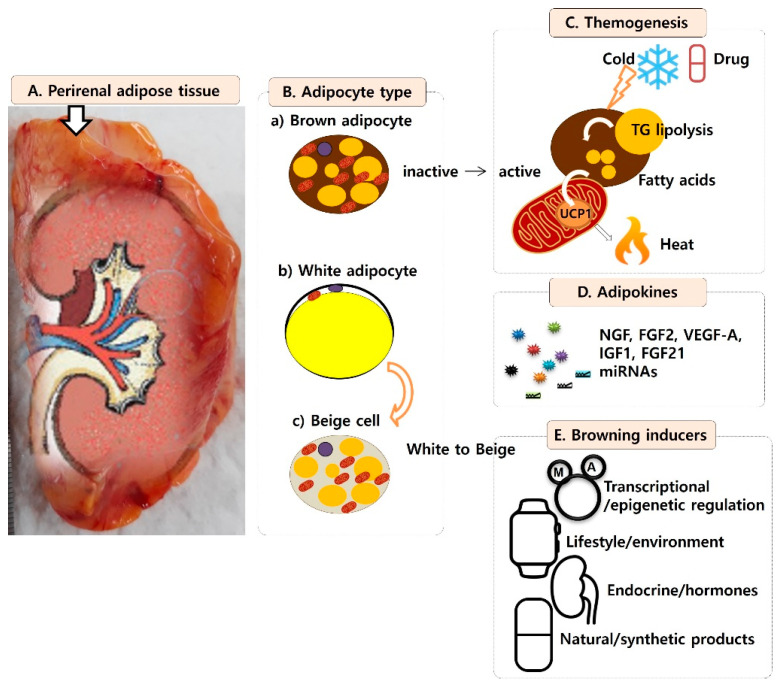
**Characterization of perirenal adipose tissue.** (**A**) Anatomic location of perirenal adipose tissue, (**B**) Perirenal adipose tissue composing adipose cell types, (**C**) Themogenesis of brown adipocyte for calorie burning, (**D**) Adipokines, secreted by brown, white and beige adipocytes, and (**E**) Browning inducers for white adipocyte transformation into beige cell.

There are three types of fat around the kidneys: paranephric fat, renal sinus fat, and perirenal fat. The paranephric fat is located outside the kidney membrane and is made up of white fat [1]. The renal sinus fat lies around the renal blood vessels and is found in the kidney membrane, and increases in proportion to obesity. Perirenal fat is located in the retroperitoneal cavity, and has been considered a simple connective tissue that protects the kidneys and renal blood vessels from external physical stimulation (Figure 1A) [1].

However, as adipose tissue has been recognized as an endocrine organ that secretes various adipokines and does not just serve for energy storage, perirenal adipose tissue has come to be regarded as a tissue that directly affects metabolic diseases, such as diabetes, obesity, and cardiovascular abnormalities [2]. In its role as an endocrine organ, perirenal adipose tissue contains a large number of brown adipose cells [3] and highly activated beige cells that are produced by the conversion of white adipose cells [4]. Thus, perirenal adipose tissue is considered to be a very useful cell source in the therapeutic aspect.

Nevertheless, all perirenal adipose tissue obtained from a healthy donor during a kidney transplant is incinerated as medical waste. To increase the possibility of its clinical application, this review paper discusses the characteristics and potential applications of perirenal adipose tissue.

## 2. Adipose Cell Types in Perirenal Adipose Tissue

Adipose cells that make up the perirenal adipose tissue are largely divided into white and brown cells, like with other adipose tissues (Figure 1B). White adipose cells store energy in the form of triglycerides, and are decomposed into fatty acids and glycerol when fasting. They affect the appetite and insulin sensitivity by secreting hormone-like molecules, such as leptin and adiponectin, in the same way as endocrine organs do [5]. Brown adipose cells, meanwhile, maintain the body temperature by releasing chemical energy as heat through the uncoupling protein 1 (UCP1)-mediated pathway, as a defense mechanism against low temperatures (Figure 1C) [6,7].

Histologically, adipose cells have a uniform shape divided by a thin collagen septum. In white adipose cells, the cytoplasm is pushed to the edge by the pressure of the fat drop. The nucleus, meanwhile, is small, thin, elliptical, and pushed to one side, with one big fat drop in the middle (Figure 1B(b)) [8]. Brown adipose cells are small and contain many fat droplets (Figure 1B(a)) [3]. When white adipose cells express high UCP1 and have many small fat droplets, they are called beige cells (Figure 1B(c)) [9]. Beige cells differ from brown adipose cells in terms of their origin, but have the same function of consuming energy as heat; thus, they are clinically valuable.

## 3. Benefits of Brown Adipose Tissue

The main role of brown adipose tissue is to keep the body temperature constant by generating heat; to generate 300 kcal, 50 g of brown adipose tissue is consumed (Figure 1C) [10]. The calorie-burning effect of brown adipose tissue can be applied to treat obesity and insulin resistance, which are metabolic diseases caused by an excessive accumulation of energy.

When a brown adipose cell is activated, glucose and fatty acids are effectively removed from the blood; blood glucose is eliminated by activation of the β3-adrenergic receptor in the brown adipose cell membrane, followed by increased synthesis of glucose transporter 1 (GLUT1), a glucose transporter, by cyclic adenosine monophosphate (cAMP) in the cytoplasm [11]. Triglycerides in the plasma are removed by the activation of lipoproteinase and CD36 secreted by brown adipose cells [12]. Thus, the activation of brown adipose cells is effective at increasing the insulin sensitivity and energy consumption, and reducing the weight.

Until recently, brown adipose tissue was thought to be nonexistent in humans at all stages of life, from infancy to adulthood. However, with the development of equipment to measure metabolic activity (fluorine-18-fluorodeoxyglucose positron emission tomography (18F-FDG-PET)/computed tomography (CT)), brown adipose tissue was discovered to be present in adults in thermo-sensitive tissues [13]. In particular, large quantities of brown adipose tissue were found around the kidneys, and its activity was high [14]. In our ongoing preliminary experiment, we reserve 302 peripheral adipose tissues; the average weight of the samples was 229.19 ± 136.53 g and the average age of the kidney donors was 32.98 ± 9.94. Using 17 samples, we measured brown fat’s distribution and found it to be present in 10–60% (*v*/*v*) of tissues. The brown fat volume showed significant individual differences.

## 4. Brown Adipose Tissue as a Heat Generator

The organelle involved in the energy generation is the mitochondria, and chemical energy and thermal energy are generated through two channels in the inner mitochondrial membrane. A proton exits the mitochondria through the electron transport path, causing a potential difference; when a proton enters through the ATP synthesis complex, chemical energy (ATP) is produced, and when a proton enters the UCP1 pathway, thermal energy is generated by the activation of fatty acid oxidation of mitochondria (Figure 1C) [15].

Brown fat is a specialized tissue that we use to adapt to the cold. When exposed to low temperatures, catecholamine (especially norepinephrine) is secreted from the sympathetic nerve, and its receptor (β3-adrenergic receptor) is activated. Then, UCP1 in the inner mitochondrial membrane is activated. As we experience regular differences in temperature, brown adipocytes are in constant activity with temperature-related genes, but beige cells derived from white adipocytes are activated only when we experience low-temperature exposure [16].

## 5. Brown Adipose Tissue as an Endocrine Organ

Activated brown adipocytes secrete substances through the endocrine pathway and affect other metabolic tissues (motor muscles) to regulate the energy metabolism [4] and inflammation [17]. The substances secreted by brown adipose tissue are called brown adipose tissue (BAT) adipokines or BATokines, which are secreted by the autocrine, paracrine, peripheral, and endocrine pathways (Figure 1D) [18].

The substances for autocrine and peripheral secretion are NGF, FGF2, and VEGF-A, which are involved in brown adipocyte growth, vascularization, neuronalization, and blood flow processes; these substances play a role in activating brown adipocytes when exposed to the cold [18]. The substances secreted by the endocrine system are IGF1 and FGF21. IGF1 plays a role in reducing the concentration of glucose in the blood [19]. FGF21 is increased in the blood by the activation of brown adipocytes when exposed to low temperatures [20], is involved in white adipocyte browning [21], and regulates energy metabolism through the lipoprotein catabolism pathway [22]. We analyzed NGF, FGF2, VEGF-A, IGF1, and FGF21’s concentrations using 10 peripheral adipose tissues. Using 25 g of each tissue as the initial volume, a stromal vascular fraction (SVF) was obtained using a manual kit (Ustem kit, Ustem Biomedical, Seoul, Korea), according to the manufacturer’s instructions. The volume of the final product was 1 mL, and NGF 3.56 ± 0.25, FGF2 230.27 ± 167.24, VEGF-A 7.50 ± 5.95, IGF1 2830.85 ± 5201.98, and FGF21 3.36 ± 0.19 pg/mL were measured. FGF2, VEGF-A, and IGF1 showed significant individual differences, while NGF and FGF21 were relatively even. 

Brown adipose tissue also plays a role in the inflammatory response. The directly secreted anti-inflammatory BATokines by brown/beige adipocytes are SLIT2-C, VEGFA, IGF-1, FGF21, CXCL14, L-PGDS, follistatin, IL6, and GDF15 [17]. In addition, when an inflammatory microenvironment is formed (e.g., obesity), the infiltration of macrophages and other immune cells into the adipose tissue is increased. Immune cells mostly secrete pro-inflammatory cytokines, which inhibit the ‘transition of white to beige adipocytes’ and promote ‘whitening of brown adipocytes’. Phenotypically whitened brown adipocytes secrete pro-inflammatory BATokines, such as Chemerin, IGF-1, CX3CL1, RBP4, TNFα, GDF8, ET-1, IL6, IL1, and MCP1 [17]. The whitening brown adipocytes have reduced thermogenic activity and inhibited energy expenditure ability, thus losing their physiological efficacy as brown adipocytes.

Brown fat is also related to the circulating exosomal miRNAs. BAT secretes exosome microRNAs to inhibit transcription. When BAT is transplanted into mice lacking the miRNA-processing enzyme dicer that makes microRNAs, various types of microRNAs are observed, glucose tolerance is reduced [23], and miR-92 is known to be related to the glucose absorption of brown fat [24].

## 6. Developmental Characteristics and Representative Markers of BAT

The perirenal adipose cells exist as adipocytes in the prenatal stage and mature after birth, and this process is called whitening [25]. This is different from the typical white adipocyte maturation seen subcutaneously; the rate of differentiation into adipocytes is faster than that subcutaneously [25] and the activity of brown adipocytes in the perirenal area is similar to that of typical brown adipose cells around the scapula [26].

The origin cells of brown adipocytes are found in the embryonic mesoderm, and among adipocytes, the cells expressing myogenic factor 5 (MYF5) differentiate into brown adipocytes and myoblasts, and then differentiate into muscle and fat, depending on the presence or absence of the PR/SET domain 16 (PRDM16) gene. As such, brown adipocytes and muscle have the same origin of development and are functionally related; thus, brown adipocyte activation is possible by exercise [27]. In addition, even adipocytes that do not express MYF5 can differentiate into beige cells when UCP1 expression occurs [28].

The main marker of brown adipocytes is UCP1, which is involved in the process of heat production by the oxidization of fatty acids through the activation of the uncoupling respiratory chain [29]. Secreted protein, acidic and rich in cysteine (SPARC), is an adipokine involved in the maintenance of brown fat, also called osteonectin. Calsyntenin 3 (CLSTN3) is involved in multiocular expression, with numerous small droplets representing a histological characteristic of brown adipocytes. Potassium two pore domain channel subfamily K member 3 (KCNK3) has a temperature-sensitive function. Peroxisome proliferator-activated receptor-gamma coactivator-1alpha (PGC-1α) and PRDM16 are brown fat transfer factors. PPARG coactivator 1 alpha (PPARGC1A) and Cbp/P300 interacting transactivator with glutamic acid [E] and aspartic acid [D] rich carboxy-terminal domain 1 (CITED1) are transcription cofactors. Retinoid X receptor gamma (RXRγ) is a differentiation factor. In addition, Ebf3, Fbxo31, Lhx8, TBX1, ELOVL3, and CIDEA are used as typical brown adipocyte markers. The human-specific brown adipocyte markers are ACOT11, PYGM, and FABP3. HMGCS2 and CKMT1A/1B show increased expression in brown adipocytes compared to white adipocytes [14,30]. Other brown/beige adipocytes and white adipocyte markers are summarized in Table 1.

When UCP1 is expressed in white adipocytes, it becomes a beige cell showing intermediate characteristics between white and brown adipocytes, and shows a temperature-sensitive phenotype in response to various stimuli, such as low temperature, drugs, or genetic factors [4]. When cells turn into beige cells, they express CD137, Tbx1 Tmem26, and Epsti1 [31], but leptin, peroxisome proliferator-activated receptor gamma (PPARγ), HOXC8, and HOXC9’s expression is decreased [14].

## 7. Main Stimulators for Activation of Brown Adipocytes

The main stimulating factors for the activation of brown adipocytes and beige cellization are low temperatures and drugs (Figure 1C) [32]. A cold temperature is the most effective inductor; when treated for a long (2 h per day for 6 weeks) or short time (6 h per day for 10 days), heat consumption is increased and body fat is significantly reduced [33]. A known mechanism for activation is non-shivering thermogenesis. The sympathetic nervous system is stimulated by the cold to activate brown adipocytes, and the hydrolyzed triglycerides produce fatty acids, generating heat [34].

Browning of white adipocytes is induced by UCP1 activation when exposed to a low temperature [5,7]. Because glucose and fatty acids are effectively consumed to generate heat, this process is considered to treat metabolic diseases. Thus, UCP1-activating drugs are being studied [4]; Mirabegron, a β3 antagonist, was originally approved as a treatment for overactive bladders, but it has been reported to increase energy consumption by activating brown adipocytes [35]. Spicy capsaicin derivatives activate temperature-related genes through the same receptor of white adipocytes’ browning [36]. Liraglutide, an antidiabetic drug, acts on the GLP-1glucagon-like peptide-1 receptor and significantly reduces the weights of obese patients by increasing their energy consumption [37]. Chenodeoxycholic acid (CDCA), a bile acid, induces brown adipocyte activation by enhancing mitochondrial respiration [38], and activates brown adipocytes by stimulating intracellular thyroid hormones through the G protein-coupled receptor (TGR5) [39].

Bone morphogenetic protein 7 (BMP7) and BMP8b are important for brown adipocyte maturation, temperature sensitivity, and browning of white adipocytes. BMP8b was found to be involved in weight loss through brown fat activation [40]. In overweight patients with type 2 diabetes, the fibroblast growth factor 21 (FGF21) analog showed decreased plasma lipids, increased blood adiponectin levels, and significantly decreased body weight [4].

As an attempted drug, 2,4-dinitrophenol, a drug similar to UCP1, was used as a weight loss drug in the 1930s, but was discontinued due to deaths from high fever and adverse effects when patients were given too high of a dose [16]. CL316,243, a β3 antagonist, also failed due to various drug receptors and poor oral activity [34].

## 8. Other Factors for Activation of Brown Adipocytes

When exposed to cold, the browning in the perirenal adipose tissue is significantly higher in women than in men [7]. In immunohistochemical staining, 33% of perirenal adipocytes in women were UCP1-positive, but in men, only 7% were [7]. In histological comparison, smaller lipid droplets were observed in women than men [7]. In women, the following process is more active than in men: cold-activated UCP1 expression increases heat generation in the mitochondria, which leads to increased energy consumption, and consequently, to adipose tissue loss [41]. These gender-specific physiological differences are related to sex hormones. The related hormones are: (1) Follicular hormone estradiol (E2), a female hormone that increases the metabolic rate in the interphase cell through E2 and induces heat generation in brown fat (when the α2-adrenergic receptor, a pathway that directly affects brown fat, is activated, the adrenergic signal is suppressed [7], and E2 activates brown adipocytes through the α2-adrenergic receptor’s inhibition); (2) Testosterone inhibits brown adipocytes’ activity by suppressing UCP1 [42]; (3) Estrogen induces brown adipocytes’ activation and white adipocytes’ browning [7]; (4) Gonadotropin and the Y chromosome inhibit UCP1’s expression in brown adipocytes [43]; and (5) The transcription and translation processes of UCP1 are epigenetically regulated according to the sex [44].

In adults, 70–80% of perirenal fat is composed of brown adipocytes [14], and brown adipose progenitor cells are distributed throughout the perirenal adipose tissue. While inactive brown adipocytes’ distribution differs depending on the location, when closer to the adrenal glands, inactive cells are increased. The inactive cell is expressed via the SPARC gene, which is a representative gene indicating the inactive state [3]. A macrophage is a new cell-type known to mediate the browning of white adipocytes [45]; previously, it was just known as a cell that secreted catecholamine. The size of BAT is opposite to obesity and age [34], while white adipose tissue is proportional [3]. Beige-ization of white adipocytes significantly decreases after the age of 40 [46].

## 9. Transformation of White Adipocytes into Beige Cells

In the resting state of the cell cycle, beige cells show gene expression similar to white adipocytes, but are stimulated by a low temperature or UCP1 expression. Beige cells consume energy similar to brown adipocytes [4]. Because of their two-sided nature, there are two hypotheses about the origin of beige cells: (1) Progenitor cell model: a beige cell is derived from a specific progenitor cell population that responds to stimuli, such as low temperatures or specific genetic regulation, and (2) Interconversion model: a beige cell comes from a mature white adipocyte and is transdifferentiated by appropriate stimulation [47]. Additionally, an ambient temperature, the genetic background, and the local location are believed to have an effect [4].

The concept of converting white adipocytes into beige cells is very useful in the therapeutic aspect to treat metabolic diseases [4]. If white adipocytes can convert to beige cells through the browning process, then histologically, a large number of small lipid droplets will be visible, and genetically, UCP1 expression can increase, becoming a cell whose purpose switches from energy storage to energy consumption.

The reported browning inducers of white adipocytes are constant low-temperature exposure, transcriptional/epigenetic regulation factors, lifestyle/environmental factors, endocrine/hormones, and natural/synthetic pharmacological products (Figure 1E). The reported temperature-sensitive factors are PGC-1α, PRDM16, MMPs, thyroid hormones, bile acids, natriuretic peptides, FGF-21, and cytokines. The hormones are irisin, tyrosine, and catecholamine. Irisin is secreted by muscles during exercise to promote browning [48], the thyroid hormone is involved in secreting irisin [49], and catecholamine, secreted from the adjacent adrenal glands, is involved anatomically [7]. The metastasis modulators are PPARγ, PRDM16, PGC-1α, and early B-cell factor-2 (EBF2) [50].

As environmental factors, diet and exercise are important for browning. The dietary compounds are capsaicin (and its analog capsinoids), menthol, 6-paradol, allyl isothiocyanates, benzyl isothiocyanates, 3,5,40-trihydroxy-trans-stilbene (a kind of polyphenol), curcumin, green tea catechins (e.g., epigallocatechin, epicatechin gallate, epicatechin), berberine, fish oil plus all-trans retinoic acid, dietary methionine, fucoxanthin, luteolin, citrulline, bile acids, resveratrol, n-3 polyunsaturated fatty acids, linoleic acid, 5-methyl-2-isopropylphenol, β-lapachone, polyphenol-rich food, and artepillin C, which has a thermogenic potential associated with UCP1 [51,52].

Physical exercise stimulates the central nervous system, especially specific neuronal populations such as agouti-related protein (AgRP) and proopiomelanocortin (POMC) neurons. POMC neuron activation stimulates browning, while the AgRP neuron suppresses it [53]. Through the POMC neurons, insulin and leptin signaling are regulated. In leptin signaling, exercise stimulates JAK2 and STAT3 tyrosine phosphorylation, which transcribe anorexigenic neuropeptides. In insulin signaling, exercise enhances IRS-1/2 and Akt activation and Fox01 phosphorylation, and sequentially halts the transcription of orexigenic neuropeptides.

The pharmacological products are PPAR-α agonist, adrenergic receptor stimulator, thyroid hormone administrator, irisin and FGF21 inducer [52], and adenylate cyclase activator (e.g., forskolin) [54]. Bioinformatics also are used to increase the pharmacologic efficiency [55]. The DNA microarray is used to quantify gene expression, RNA sequencing is used to quantify RNA expression, and chromatin immunoprecipitation with sequencing (ChIP-seq) is used to identify protein-binding sites in DNA and examine histone modifications. For example, the white adipocyte gene expression profiles of normal mice and transgenic mice overexpressing EBF2 were compared by RNA sequencing. The mice overexpressing EBF2 in white adipocytes showed a brown adipocyte genotype, and white adipocyte-specific gene expression was decreased when compared to the normal mice.

## 10. Transplantation of Brown Adipocytes

Transplantation of brown adipocytes into diabetic or obese mice resulted in significantly lowered blood glucose levels, systemic inflammation, and concentration of serum adipokines [56]. When brown adipocytes were transplanted into IL-6-deficient mice, the concentration of IL-6 in the body increased, and insulin sensitivity in the skeletal muscle and adipose tissue was increased. This result indicates that IL-6 was secreted from the implant, and although IL-6 is a proinflammatory cytokine, it has the effect of increasing insulin sensitivity in the skeletal muscle and adipose tissue [56]. Meanwhile, temperature-related gene expression was not changed, which means that transplantation of brown adipocytes has no sensitivities to the temperature pathway [57]. Up to now, human transplantation of brown adipocytes has not been attempted because the safety of this has not been confirmed.

## 11. Renal Pathological Aspect

The advantages of perirenal adipose tissue, as described above, are limited to healthy donor tissue. Because perirenal adipose tissue is anatomically in direct contact with the kidneys and adrenal glands, when the physical size increases due to obesity or other problems, this can lead to various pathological abnormalities [58].

The size increase of the perirenal adipose tissue means an increase of white adipocytes that (1) secrete inflammatory adipokines, (2) increase the free fatty acids, glucose, triglycerides, and uric acid, (3) decrease the blood flow in the renal artery and renal parenchyma, (4) decrease the glomerular filtration rate, (5) increase the sodium reabsorption, and (6) stimulate renin secretion, which causes acute/chronic renal failure [59]. In addition, adipose afferent reflex, renin-angiotensin-aldosterone system activation, and adipokine/cytokine elevation are associated with hypertension, cardiovascular disease [60], atherosclerosis [61], and insulin resistance [62]. Also, dormant brown adipocyte activation and pro-inflammatory cytokine synthesis are associated with tumor progression. Therefore, it is necessary to consider the pathological risk of perirenal adipose tissue when obtained from an unhealthy donor.

## 12. Conclusions

The perirenal adipose tissue contains a large number of brown adipocytes and there is high conversion efficiency of beige cells from white adipocytes. Technically, we have identified the stimulating factors for inactive brown adipocytes, and browning factors have also been also identified. This research has found that adipocytes of the perirenal adipose tissue obtained from a healthy donor represent an effective human cell source with which to treat metabolic diseases through energy consumption, rather than being incinerated as medical waste. 

The approximate benefits of peripheral adipose tissue were summarized in Table 2 comparing subcutaneous adipose tissue.

## Figures and Tables

**Table 1:** Representative markers for distinguishing white and brown/beige adipocytes.

Adipocyte Type	Representative Marker
White	LEPTIN, HOXC8, HOXC9, NAT8L, PRLR, NRG4, TMEM120B, ADRB3, AQP7, GPR81, SLC27A1/FATP1, SLC7A10/ASC-1, NRG4, AQP7, ASC-1
Brown/Beige	UCP1, SPARC, CLSTN3, KCNK3, PGC-1α, PRDM16, PPARGC1A, CITED1, RXRγ, EBF3, FBXO31, LHX8, TBX1, ELOVL3, CIDEA, ACOT11, PYGM, FABP3, HMGCS2, CKMT1A/1B, GPR119, PAT2, ZIC1, LEP, TMEM26, P2RX5, TNSFRSF9, SHOX2, MPZL2

**Table 2:** Comparison of approximate benefits of subcutaneous and peripheral adipose tissues.

Contents	Subcutaneous Adipose Tissue	Perirenal Adipose Tissue
Donor health status	mostly overweight	mostly healthy
General tissue acquisition path	cosmetic liposuction	kidney transplant
Anatomic tissue location	subcutaneous	viscera, especially perirenal
Relevance to metabolic diseases	symptom aggravation	symptom relief
Dominant cell type	white adipocyte	brown adipocyte
Secreted adipokine properties	multiple pro-inflammatory	multiple anti-inflammatory
Brown adipose cell content	little	numerous
Brown adipose cell distribution	scattered	close to the adrenal gland
Brown adipose cell state	dormant	active
UCP1 gene activity	low	high
Conversion rate into beige cells	rare	frequent
Beige cell activity	low	high
Energy consumption efficiency	low	high

## Data Availability

Not applicable.
